# Supplementary material for: Novel participatory methods for co-building an agent-based model of physical activity with youth
Source: PLoS One. 2020 Nov 10;15(11):e0241108. doi: 10.1371/journal.pone.0241108 (PMC7654780; doi:10.1371/journal.pone.0241108)
Supplement: S1 Appendix — (DOCX) [file pone.0241108.s001.docx]

**Graphs Over Time for Agent-Based Models**

***Adding spatial and social notations***

| **Context** | At the beginning of a group model building session as it is a springboard for discussion about the problem to be modeled |
| --- | --- |
| **Purpose** | To engage participants in a group model building session in framing the problem, initiating mapping, eliciting variables (including important social and spatial variables to consider) and gathering input in deciding the reference modes for the study. |
| **Nature of group task** | Divergent |
| **Time** | Preparation time: 15-30 minutes  Time required during session: 45 minutes  Follow-up time: 15-45 minutes |
| **Materials needed** | - Pencils and erasers (to sketch trends and enable changes) - Pens - Colored markers - Flip sheet paper with axes labeled:   X-axis: appropriate time scale (e.g., hours of the day)  Y-axis: appropriate variable (e.g., physical activity)   - - Small sticky notes / labels in 3 colors |
| **Inputs** | None |
| **Outputs** | - - Aggregated graphs over time to assess patterns   - Candidate variables for the model |
| **Roles** | Facilitator: works with the group and has some experience with dynamic modeling  Modeler: listens to what is being graphed and the way people are talking about the graphs. They must also be able to conceptualize early seeds of system structure.  Wall builder (optional): clusters graphs and talk about themes  Runner (optional): bring the graphs from the community facilitator if the group is large  Recorder: documents the session and photograph the clustered graphs |
| **Steps** | 1. Introduction of graphs over time    1. Facilitator introduces the concept of graphs over time. Example script follows for physical activity that can be adjusted as needed for other topics:   🡪 *Last session we talked about what makes you more or less likely to be active. Today we want to talk about how your physical activity changes throughout the day – this is what our final model will actually involve!* 🡪 *On your sheet, you can see the start of a graph. The bottom axis represents time throughout a day. We’ve started at 6am and gone until midnight.*  🡪 *The axis on the left represents your physical activity. To help with the scale, we’ve given four categories. The bottom says ‘sedentary’ which represents when you’re not moving around – for example, just lying on the couch.*  🡪 *Then it moves up to light activity, which is activity that doesn’t really make your heart beat faster or make you sweat – but is movement. This could be something like when you walk in between classes.* 🡪 *Next is medium activity, which is when you can feel your heart beating but are not totally out of breathe or super sweaty.*  🡪 *The highest part of the left axis is difficult activity, which is activity that makes you breathe really hard and sweat.* 🡪 *You all will use these graph sheets to draw your physical activity throughout the day. Let’s go through an example first.*   - 1. Facilitator walks through an example. Considerations for the example: 1) potentially make the example unrealistic so it can’t be directly copied or lead to bias (e.g., lots of ups and downs, someone running a marathon). 2) make explicit mention that we are looking for general patterns, and that the graph does not need to be perfect. 3) facilitator can consider making a mistake during their example to model that mistakes are ok!  1. Participants sketch initial trends    1. Participants can be instructed to use pencils to sketch their initial trend that they can finalize in markers. Example script follows for physical activity that can be adjusted as needed for other topics: 🡪 *Now, try to do this for a typical weekday on your big piece of paper. Don’t worry if it’s not perfect – your best approximation is fine! You can use pencils first and then trace over your final line with a marker.*    2. Facilitator can move among the participants and answer questions and offer help. Some specific considerations to the example of physical activity follow that may be helpful for other topics: 1) If participants are having trouble defining what typical is, they can be prompted to sketch ‘yesterday’ (unless yesterday was unusual), 2) If there are days that participants usually do physical activity – e.g., practice on Monday/Wednesday – then request that participants use one of those days, rather than a day they do not do physical activity. 2. What & Where – Adding Activity and Spatial aspects    1. Once all participants are done drawing their graphs, instruct them to add information on what they are doing and where they are to their graph. Example script follows for physical activity that can be adjusted as needed for other topics: 🡪 *For your lines, we now want you to note* ***what you’re doing and where you are*** *throughout the day. This will help us know what the characters in our model are doing and help us allow the ‘characters’ in our model to move around an environment.* 🡪 *We want you to note what you’re doing both when you’re being sedentary, and when you’re being active. If you sometimes do one thing and sometimes another, you can note both of those on here.*   🡪 *Take the [COLOR 1] sticky notes and stick them on your graph to note what you are doing at that point. We want you to note what you are doing when you are being active, and what you are doing when you are not being active. Try to be as specific as possible.*  🡪 *Take the [COLOR 2] sticky notes and stick them on your graph to note* ***where you are*** *throughout the day. Try to be as specific as possible.*   - 1. Facilitator walks through an example. Considerations for the example: 1) Include an example where there are multiple options for “where” based on the graph, and indicate that all locations should be noted. For example, if someone goes to yoga, or plays tennis, or walks in the evening, they should note all of those in step 1 and then note all of the locations in step 2.   2. Youth add their own labels.  1. Who: Adding Social aspects    1. Once all participants are done annotating the ‘what’ and ‘where’ aspects of their graphs, instruct them to add information on what they are doing and where they are to their graph. Example script follows for physical activity that can be adjusted as needed for other topics: 🡪 *And last but not least, you are probably with some other people throughout the day in these different places. You don’t have write people’s names – you can use: girlfriend/boyfriend; friends; family – mom/dad/sister/brother/etc.; classmate that isn’t a friend; coworker if you have a job; teammates; or others. Since there will be lots of characters in our model, we want to make sure they interact correctly.*   🡪 *Take the [COLOR] sticky notes and stick them on your graph to note* ***who you are with*** *throughout the day. Try to be as specific as possible, and make sure to note when you are alone.*   - 1. Facilitator walks through an example   2. Youth use pens and one color of sticky note to do this themselves  1. As needed, steps 1-4 can be repeated with different time frames. For example, physical activity patterns tend to vary between weekday and weekends so the above steps may need to be done once for a typical *weekday* and separately for a typical weekend day.   There are two options that can be adapted and either or both followed for sharing and synthesizing the graphs:  Option #1   1. Have the participants share their graphs. 2. As the participants share, a facilitator and/or wall builder try to meaningfully cluster graphs and themes that arise 3. The facilitator and/or wall builder summarizes the clusters and themes and guides the participants to discuss   Option #2  6. After the session, graphs can be input into spreadsheets and digitized to visualize patterns across and aggregated over participants.  7. At a subsequent session, present the digitized graphs and summarize key patterns that arise  8. Guide the participants in a discussion of the patterns and the problem they imply. |

**Mapping Important Locations**

| **Context** | Beginning of a modeling project that requires understanding spatial context and/or geographic movement. |
| --- | --- |
| **Purpose** | To elicit important/relevant geographic locations and potential movement between them. |
| **Nature of group task** | Divergent/Convergent |
| **Time** | ~60-120 minutes |
| **Materials needed** | - Large, poster-sized map of relevant location - Appropriate tools to hang the map - Smaller maps (e.g., flip chart paper) for each individual - Colored markers and highlighters (variety of colors for each participant) - Stickers (e.g., stars, arrows) - Tape/sticky tack |
| **Inputs** | - Graphs Over Time (generally should be completed prior to this script) - Participant pictures (requested during a prior session) of relevant locations.   For example, for physical activity related model, ask participants in prior session, “During our next session, we’re going to keep talking about different locations and how that relates to your movement and physical activity. For next time, please take pictures of roughly 5 important places that we should include in our story. Try to get a variety of places – places that help you to be active and/or places that make it harder to be active. Or, places where physical activity happens a lot, and places where it doesn’t.” |
| **Outputs** | Aggregate information from participants about relevant locations that can be used to:   - identify geographic boundaries for an ABM - important locations to include in the model - help specify geographic movement to build into the ABM |
| **Roles** | Facilitator: works with the group and has familiarity with the language of the group, good facilitation skills  Scribe/Facilitator**:** who is familiar with maps/spatial awareness and can help take group notes and attach things to map  Recorder: who has exposure to ABM who can take notes on the discussion |
| **Steps** | 1. Introduction of mapping activity 2. The facilitator is at the front of the room where the poster-sized map is tacked to the wall. Participants are either sitting individual or in small groups with a copy of the map and their pictures (from prior session assignment). 3. The facilitator begins by explaining that “we’re going to spend the next hour doing a mapping exercise using the pictures you took” 4. The facilitator explains that the goal of the mapping activity is to identify locations that are shared and important for participants in relation to the concept of interest (e.g., physical activity) in order to define what locations to include in the model. 5. The facilitator then orients the participants to the map by going over basics (map boundaries, directionality, etc.) and by highlighting well-known’ locations (e.g., school, Walmart, etc.). (on big map) 6. The facilitator then further orients the participants by providing an example and locating an individual’s (e.g., a community/local facilitator’s) home. (on big map) 7. Transition to orient the participants to their small printed maps 8. The facilitator asks the individual participants to all locate their home residence on their individual map. The facilitator provides the participants time to find their locations and he/she and other facilitators go around the room to assist as necessary. After participants find their home location on their individual map, they should note their home location on the large map. 9. The facilitator then asks the individual participants to spend 10-15 minutes to locate where their pictures were taken on their individual maps (placing dots with markers or using stickers). Participants should be encouraged to talk to other participants (e.g., in small groups at tables). They can be encourage to discuss what about the places they identified was important. 10. Group discussion and summarize key themes 11. Facilitator should ask the participants to share in the large group, round robin style, 1 place where physical activity happens and 1 place where it doesn’t. This continues until all pictures have been shared. 12. The facilitator should summarize key places and engage participants in a brief discussion about what places are more relevant for the issue of interest and why.   4. Mobility between places (Optional – depending on issue of interest and relevance to modeling problem)   1. Facilitator transitions the group to thinking about mobility between places, e.g., “We identified many important places, but we know that we move between these in different ways. Our next activity is to better understand our movement from one place to another.” 2. The facilitator asks each individual to translate their Graph Over Time into pathways on the map (for a usual weekday). The facilitator should first highlight an example on the larger map, narrating an obviously fictional account of their movement. 3. Individuals should then be provided with about 10-15 minutes to create an annotated map of their pathways using highlighters and notes to describe important points about their movement (e.g., “In the morning I walk from home to school”; “I go to the park after school to play some basketball with my friends”)   5. Group discussion and summarize themes   1. The facilitator asks the participants to each share their pathway. 2. Each subsequent participant is asked if they had anything in common with previous pathways and to add any information that is different. 3. The recorder should take careful notes about commonalities and differences that arise about the pathways themselves (e.g., driving versus walking from home to school) 4. The facilitator should summarize themes and engage participants in discussion about commonalities and differences in locations and mobility patterns |

**State Chart Review**

| **Context** | Beginning of a modeling project after some initial work has been done to identify key variables and patterns of interest |
| --- | --- |
| **Purpose** | To summarize key states of interest, to clarify fuzzy transitions or capture additional information about model structure, to elicit feedback from participants about model states and structure |
| **Nature of group task** | Convergent |
| **Time** | ~30 minutes |
| **Materials needed** | - Screen, projectors or large poster of draft state chart - Handouts for participants reflecting state charts of model agents - Highlighters |
| **Inputs** | Seed structure(s) reflecting the state charts of the agents |
| **Outputs** | Refined state charts for model |
| **Roles** | Facilitator: works with the group and has familiarity with agent-based models  Recorder: who has exposure to ABM who can take notes on the discussion |
| **Steps** | 1. Introduction of state chart review activity 2. The facilitator is at the front of the room where the draft state chart is presented (either poster-sized or projected). Participants are either sitting individual or in small groups with a copy (or copies) of the state charts. 3. The facilitator begins by explaining the basics of state charts as a basic building block of the model 4. The facilitator explains how the drafted state chart was created (e.g., “we used information we gathered from prior sessions to identify key states that participants will likely move to and from”) 5. The facilitator explains that the goal of the activity is to get the participants feedback on the appropriateness and quality of the drafted state chart. 6. Participants trace pathways on the state charts 7. The facilitator asks the individual participants to use a highlighter and trace their potential pathways through the states and identify if transitions or states are missing or wrong. 8. Participants are provided 5-10 minutes for tracing their pathways 9. Group discussion and summarize key themes 10. Facilitator convenes the group and asks participants to share their pathways with the large group, round robin style, identifying if there were states or transitions missing. 11. Recorder takes notes on the identified refinements to the state chart 12. Facilitator recaps key additions and changes to the state chart indicated by the discussion. |

**Interviewing to Understand Decision Rules**

| **Context** | Beginning of a modeling project after initial work has been done to identify key system structure for the model |
| --- | --- |
| **Purpose** | Eliciting variables and factors that influence transitions between important states for a model |
| **Nature of Task** | Divergent |
| **Time** | Total: 75-135 minutes (Phase 1: 45-75 minutes; Phase 2: 30-60 minutes) |
| **Materials** | 1. Projector with software or butcher/chart paper and markers 2. Paper and writing utensils for note-taking during interviews 3. Recorder’s materials |
| **Inputs** | Guides for interview questions  Seed structure(s) reflecting the state charts of the agents |
| **Outputs** | State chart with influences/impacts mapped onto the transitions that reflect if how those influences trigger (or hinder) state shifts |
| **Roles** | Modeler/Facilitator #1: with expertise in ABM and underlying decisions rules of state shifting in ABM  Facilitator #2: who understands the community/participants language who can help role-play an interview  Recorder: with exposure to ABM who can take notes on the discussion |
| **Steps** | Phase #1: Interviewing   1. Introduce the concept of interviewing for research 2. Facilitator introduces that the purpose of the next activity is to gain additional information about what influences key transitions or changes related to the issue of interest. 3. Two facilitators co-introduce the concept of interviewing for research, indicating that we are going to ask the participants to interview each other in pairs 4. The facilitators role-play a mock interview for the participants 5. Following the role-play, facilitators engage the participants in a discussion about what they observed and best practices for interviewing (e.g., interviewer provides neutral responses, questions are open-ended and not leading, interviewer allows for pauses and silence, interviewer recaps and confirms their interpretation). 6. Participants (in pairs) interview each other 7. Provide participants with interview guides 8. Participants should find separate quiet areas and take approximately 30 minutes to interview each other 9. Group sharing 10. Facilitator/s convene the group and ask all to share what they learned about their interview partner 11. Facilitator summarizes key themes across the participants 12. Recorder takes careful notes about all factors highlighted with attention to their influence on decision rules   Phase #2: Annotate State Charts   1. Recap and introduce state charts 2. The modeler, who is sitting with a laptop connected to a data projector, and the facilitator are at the front of the room. The modeler could also be drawing the structure by hand on a white board or paper, as long as it is visible to the entire group. 3. The facilitator begins by explaining, “We’re going to spend the next 45 minutes or so doing a mapping exercise what influences you to do PA at different times of day. We are going to begin our discussion thinking about what we learned from interviewing each other.” The facilitator then recaps the themes and highlights from the prior interview phase. 4. The modeler then introduces the seed structure, using a state chart. For example, the states could be “Doing Physical Activity” or Not Doing Physical Activity” (Note: If changes are suggested or needed to the state chart itself, the facilitator affirms the changes while the modeler captures the changes.) 5. Ask participants to help annotate the state chart with factors that impact transitions 6. The facilitator provides an example of things that impact the state shifts, focusing on things that encourage you to stop, start, or stay in the state you’re in. (Example: What would make you do PA before school?, Example: What would be make you stay sedentary?, Example: What would make you stop doing PA once you started?) 7. The facilitator explains that participants can talk about their own experience or what they see in their peers in the community. 8. The recorders document working definitions used for key words. 9. As someone suggests something, the modeler draws the link on the model in front of the room. The facilitator and modeler will then encourage participants to add variables and relationships. The modeler tries to get things recorded using exactly the same terms as the participants. 10. Meanwhile, the recorders are taking notes on the variables named, relationships being described, and quotes or stories that help put some context around the story. 11. The process continues for each distinct context in which the state transitions may be influenced by different factors. |
